# Changes Editorial

**Published:** 1854-03

**Authors:** 


*Changes Editorial.* — Dr. Samuel R. Hollingsworth has succeeded Messrs. Francis Gurney Smith, and John B. Biddle, M. D., in the editorial management of the Philadelphia Medical Examiner.

The Examiner has long sustained an excellent character among our periodicals, and we trust that Dr. Hollingsworth will well maintain the arduous *role* he has taken upon himself.

Dr. John Dawson has undertaken the editorship of the Ohio Medical and Surgical Journal, made vacant by the death of Prof. Howard. We wish him every success in his new field of labor.

The New Jersey Medical Reporter, one of the best of our exchanges, and which has increased in size and improved otherwise very rapidly under the editorial care of Dr. Joseph Parrish, has now been transferred to Dr. G. M. Butler. The cause of this change is Dr. Parrish's removal to Philadelphia. Dr. Parrish does not, however, entirely disconnect himself from the Reporter, and his monographs will occasionally appear in it. Just now he is writing an excellent series of papers on the "Change of Life" in women. H.

